# Residential Racial Segregation in Aortic Stenosis Diagnosis and Transcatheter Aortic Valve Implantation Among Medicare Patients

**DOI:** 10.1016/j.jacadv.2023.100415

**Published:** 2023-07-19

**Authors:** Jonathan Sevilla-Cazes, Zaid I. Almarzooq, Ashley N. Kyalwazi, Yun Wang, Yang Song, Wayne B. Batchelor, V. Antoine Keller, Jordan Strom, Rishi K. Wadhera, Robert W. Yeh

**Affiliations:** a Division of Cardiovascular Medicine, Beth Israel Deaconess Medical Center, Boston, Massachusetts, USA; b Harvard Medical School, Boston, Massachusetts, USA; c Division of Cardiovascular Medicine, Brigham and Women's Hospital, Boston, Massachusetts, USA; d Boston Deep Data, Boston, Massachusetts, USA; e Inova Heart and Vascular Institute, Falls Church, Virginia, USA; f Division of Cardiology, Duke University, Durham, North Carolina, USA; g Heart and Vascular Center at Ochsner Lafayette General Hospital, Lafayette, Louisiana, USA

**Keywords:** aortic stenosis, racial disparities, surgical aortic valve replacement, transcatheter aortic valve implantation

## Abstract

**Background:**

Transcatheter aortic valve implantation (TAVI) rates are lower among Black compared with White individuals. However, it is unclear whether racial residential segregation, which remains common in the United States, contributes to observed disparities in TAVI rates.

**Objectives:**

The purpose of this study was to evaluate the association between county-level racial segregation, and aortic stenosis (AS) diagnosis, management, and outcomes.

**Methods:**

We identified Black and White Medicare fee-for-service beneficiaries age ≥65 years living in metropolitan areas of the United States (2016-2019). Using the American Community Survey’s Black-White residential segregation index, a measure of geographic racial distribution, we determined segregation in each beneficiary’s county of residence. Using hierarchical modeling, we determined the association between racial segregation and rates of AS diagnosis, TAVI receipt, and 30-day clinical outcomes (mortality, readmission, stroke).

**Results:**

There were 29,264,075 beneficiaries, of whom 22% lived in a high-segregation county. Among Black beneficiaries, high-segregation county residence was associated with decreased rates of AS diagnosis (OR: 0.97; 95% CI: 0.96-0.98) and TAVI (OR: 0.89; 95% CI: 0.86-0.93) compared with low-segregation county residence. In contrast, among White beneficiaries, high-segregation county residence was associated with higher rates of AS diagnosis (OR: 1.02; 95% CI: 1.02-1.03) and no differences in TAVI (OR: 1.00; 95% CI: 0.99-1.00). Segregation and race were not independently associated with 30-day mortality.

**Conclusions:**

Among Black Medicare fee-for-service beneficiaries, living in a high-segregation county was independently associated with decreased rates of AS diagnosis and TAVI, an association not seen among White beneficiaries. Residential racial segregation may contribute to racial disparities seen in AS care.

The development of transcatheter aortic valve implantation (TAVI) has revolutionized the treatment of aortic stenosis (AS) by offering an alternative to surgical aortic valve replacement (SAVR) with comparable or improved outcomes.^1^ Yet, numerous studies have found lower rates of aortic valve replacement (AVR), both TAVI and SAVR, among historically disadvantaged and marginalized populations, particularly Black patients.^2^^,^^3^ This has raised significant concerns regarding the equitable distribution of novel technologies. Structural racism is a major driver of racial disparities in health outcomes,^4^ but little is known about its impact on access to new cardiovascular therapies, such as TAVI. A better understanding of the factors contributing to racial disparities, including the role of structural racism on diagnosis and management of AS, is critical to promote health equity.

One of the most evident forms of structural racism is residential segregation, which disproportionately affects Black individuals in the United States.^5^ Residential segregation impedes social mobility, access to education and employment, and has been associated with adverse cardiovascular health.^6^, ^7^, ^8^, ^9^ In addition, health system segregation persists due in part to residential segregation, with outpatient practices and hospitals that serve a high proportion of Black adults being under-resourced and financially constrained.^10^ These factors may have major implications for the diagnosis of common cardiovascular conditions like AS, and the delivery of novel cardiovascular therapies such as TAVI, for Black adults. However, the extent to which the intersection between residential segregation and race is associated with disparities in the diagnosis, management, and outcomes of AS remains unknown.

In this study, we aimed to answer the following questions. 1) Is county-level Black-White residential segregation associated with rates of AS diagnosis and TAVI? 2) Does the association between county-level residential segregation and rates of diagnosis and treatment of AS differ for Black compared with White individuals? 3) Are county-level residential segregation and race associated with differences in outcomes after TAVI?

## Methods

### Study population

Using the Medicare Provider Analysis and Review (MedPAR) files and institutional outpatient claims from the Centers for Medicare and Medicaid Services, we identified all Medicare fee-for-service (FFS) beneficiaries enrolled between January 1, 2016 and December 31, 2019. We included individuals who were 65 years or older and had a self-reported race of either Black or White. To control for previously identified urban/rural disparities in TAVI,^11^^,^^12^ we limited the sample to those living in metropolitan statistical areas, based on county of residence during the study period, defined as urban clusters of at least 50,000 people in accordance with the Office of Management and Budget definition.^13^ Beneficiaries who lived in a county for which the American Community Survey’s residential segregation index (SI) was unavailable,^14^ or who had missing county data were excluded.

Patient demographics including age, sex, self-reported race, and dual Medicare-Medicaid enrollment were obtained from MedPAR files. Beneficiaries were considered dual Medicare-Medicaid enrolled if they were also enrolled in Medicaid for at least 1 month during a given year.^15^ Using data from the Centers for Medicare and Medicaid Services 2015 to 2019 Chronic Conditions Data Warehouse, we identified the presence of 67 chronic conditions for each individual in the study sample.^16^^,^^17^ The study protocol was reviewed and deemed exempt by the Beth Israel Deaconess Medical Center Review Board, given the use of deidentified patient information.

### Black-white county segregation

Our main exposure of interest was the degree of Black-White segregation in the beneficiaries’ county of residence, which we determined using the American Community Survey’s residential SI.^14^ This validated SI measures how evenly Black and White residents are distributed geographically and can range from 0 (complete integration) to 100 (complete segregation).^18^ This measurement is only available for counties with a Black population >100 (65% of counties). We dichotomized counties into high-segregation or low/moderate-segregation based on having a SI ≥60 or <60 respectively, a commonly used threshold.^9^^,^^19^

### Outcomes

We identified beneficiaries with a diagnosis of AS using International Classification of Diseases-10th Revision (ICD-10) diagnostic codes (I35.0, I35.2, I06.0, I06.2, Q23.0) in outpatient and inpatient encounters as either a primary or a secondary diagnosis.^20^ We validated the performance of these codes using a transthoracic echocardiographic report dataset linked to Medicare FFS claims and found a sensitivity of 50% and a specificity of 96% for identifying any AS.^21^ The diagnostic performance of these codes was similar regardless of county segregation (Supplemental Table 1). For those beneficiaries with more than one AS diagnostic code, only the first code was counted. Using ICD-10 procedure codes, we identified beneficiaries who had undergone an AVR with either TAVI (02RF38H, 02RF38Z, 02RF3JH, 02RF3KH, 02RF37H, 02RF37Z, 02RF3JZ, or 02RF3KZ) or SAVR (02RF07Z, 02RF08Z, 02RF0JZ, 02RF0KZ).^22^ For those who underwent multiple AVRs, only the first procedure was included in our analysis.

Our primary clinical outcome of interest was the 30-day composite of all-cause mortality, ischemic stroke, and all-cause readmission. Mortality and readmission data was obtained from MedPAR files. Ischemic stroke was defined by previously used ICD-10 diagnostic codes (I63.X, I65.X, or I66.X).^23^ Our secondary clinical outcomes were the individual components of the composite. Patients who were alive and disenrolled from Medicare within 30 days AVR were excluded from the outcomes analysis.

### Statistical analysis

We followed the guidelines for cohort studies as described in the Strengthening the Reporting of Observational Studies in Epidemiology Statement.^24^ We described the demographic and clinical characteristics of our study population, stratified by county segregation, using means and SDs, or counts and proportions as appropriate. We calculated the annual rate of AS diagnosis, TAVI, SAVR, and any AVR per 100,000 patient-years. Clinical outcomes were reported as percentages.

We fit a mixed-effect regression to model the rates of AS diagnosis, TAVI, SAVR, and any AVR among all beneficiaries, as a function of county segregation (high vs low/moderate) controlling for beneficiary demographic and clinical characteristics. To assess whether Black adults in high-segregation counties were less likely to be diagnosed with AS or receive AVR than White adults, we also included an interaction term for race and segregation in the models. To account for potential secular trends in outcomes, we included an ordinal time variable, ranging from 0 (year 2016) to 3 (year 2019) in the models. Similar models were used to assess the association between race, racial segregation, and clinical outcomes among those who underwent TAVI, SAVR, and any AVR.

As sensitivity analyses, we repeated the models describe above using SI as a discrete variable composed of 10-point increments. We also repeated these models limited to the cohort of beneficiaries with an established diagnosis of AS. A 2-sided α of 0.05 was used for all statistical tests. All statistical analyses were performed using SAS (version 9.4, SAS Institute).

## Results

A total of 29,264,075 unique Medicare FFS beneficiaries living in metropolitan areas between 2016 and 2019 were included in the analysis. Among all beneficiaries, 6,496,554 (22%) lived in a high-segregation county. Compared to beneficiaries living in low/moderate-segregation counties, those in high-segregation counties were more likely to be female (56.1% vs 54.8%), Black (14.5% vs 8.1%), dually enrolled in Medicaid (11.5% vs 8.7%), and have higher rates of comorbidities (Supplemental Table 2). Black patients with an AS diagnosis had higher rates of dual enrollment in Medicaid than White patients (29.6% vs 8.8%); however, dual enrollment rates among Black patients was similar regardless of county segregation. The demographic and clinical characteristics, stratified by race and county segregation, for those with an AS diagnosis can be found in Table 1.

**Table 1:** Baseline Characteristics of Patients With an AS Diagnosis Stratified by Race and Segregation

	White				Black			
	Overall (N = 822,265)	Low/Moderate Segregation (SI <60) (n = 636,434)	High Segregation (SI ≥60) (n = 185,831)	*P* Value	Overall (N = 42,247)	Low/Moderate Segregation (SI <60) (n = 27,753)	High Segregation (SI ≥60) (n = 14,494)	*P* Value
Age, y	80.3 ± 8.4	81.3 ± 8.1	81.9 ± 8.2	<0.0001	80.3 ± 8.4	80.1 ± 8.3	80.8 ± 8.5	<0.0001
Female	63.6	48.8	50.8	<0.0001	63.6	62.5	65.6	<0.0001
Dual enrollment	8.8	8.4	10.1	<0.0001	29.6	29.6	29.6	0.94
Myocardial infarction	0.8	0.8	0.8	0.88	1.1	1.1	1.1	0.93
Atrial fibrillation	12.8	12.9	12.6	0.0003	6.7	7.0	6.3	0.004
Chronic kidney disease	22	22.2	21.1	<0.0001	29.7	30.5	28.3	<0.0001
Chronic obstructive pulmonary disease	10.5	10.6	10.2	<0.0001	10.4	10.4	10.4	0.95
Congestive heart failure	17.8	17.7	18.1	<0.0001	22.6	22.5	22.7	0.75
Diabetes	22.7	22.8	22.6	0.04	32.2	33.0	30.8	<0.0001
Ischemic heart disease	31.2	31.4	30.5	<0.0001	29.4	29.9	28.6	0.005
Stroke/transient ischemic attack	3.5	3.5	3.4	0.03	4.7	4.6	4.9	0.15
Cancer	7.2	7.2	7.2	0.35	8.0	8.0	7.9	0.74
Hyperlipidemia	39	39.5	37.4	<0.0001	37.1	38.4	34.5	<0.0001
Hypertension	48	48.4	46.6	<0.0001	52.4	53.3	50.5	<0.0001
Obesity	19.1	19.1	18.8	0.005	24.6	24.5	24.8	0.46
Peripheral vascular disease	24.9	24.5	26.5	<0.0001	29.2	27.9	31.8	<0.0001
TAVI	6.3	6.5	6.0	<0.0001	4.0	4.3	3.5	<0.0001
SAVR	4.3	4.5	3.9	<0.0001	2.5	2.5	2.4	0.36
Any AVR	10.7	10.9	9.9	<0.0001	6.5	6.9	5.9	<0.0001

Values are mean ± SD or %.

AS = aortic stenosis; AVR = aortic valve replacement; SAVR = surgical aortic valve replacement; SI = segregation index; TAVI = transcatheter aortic valve intervention.

### Diagnosis of AS

**Figure 1:**
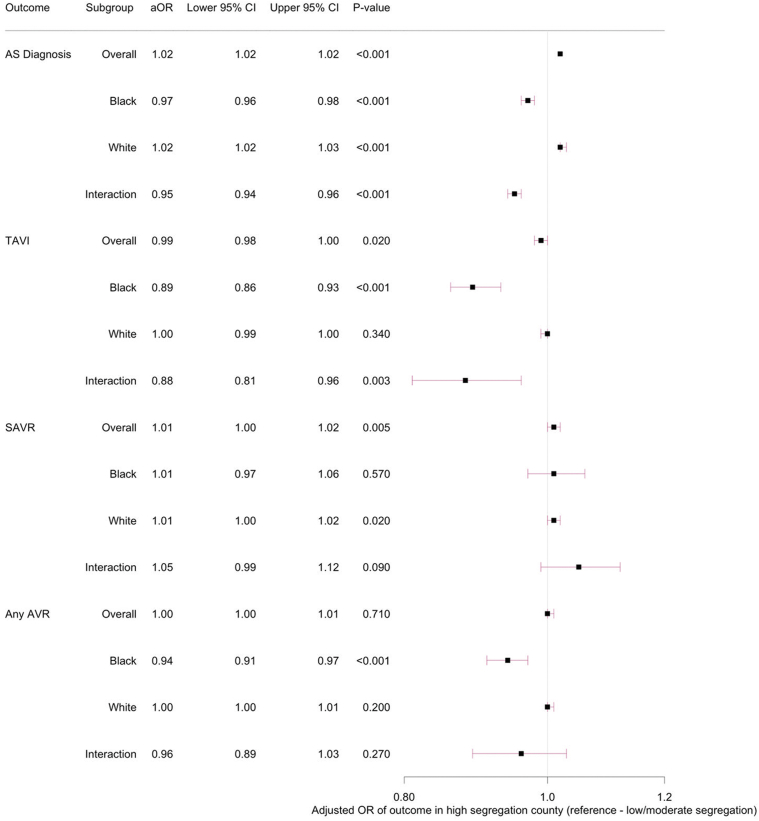
**Association Between County Segregation and AS Diagnosis/Management Stratified by Race** A mixed-effect regression was used to model the rates of aortic stenosis diagnosis, transcatheter aortic valve implantation, surgical aortic valve replacement, and any aortic valve replacement, as a function of county segregation adjusting for beneficiary demographic and clinical characteristics. For Black beneficiaries, living in a high-segregation county, compared to a low/moderate-segregation county, was associated with lower likelihood of aortic stenosis diagnosis and transcatheter aortic valve implantation. For White beneficiaries, living in a high-segregation county, compared to a low/moderate-segregation county, was associated with increased likelihood of aortic stenosis diagnosis and no difference in transcatheter aortic valve implantation. aOR = adjusted odds ratio; AS = aortic stenosis; AVR = aortic valve replacement; SAVR = surgical aortic valve replacement; TAVI = transcatheter aortic valve implantation. Interaction = Black race ∗ high-segregation interaction variable.

Among all beneficiaries, 864,512 (3%) had a diagnosis of AS during the study period. The annual rate of AS diagnosis per 100,000 patient-years was 1,083 for the overall cohort and 1,149 for those living in a high-segregation county (Table 2). In adjusted models, living in a high-segregation county, compared with a low/moderate-segregation county, was associated with an increased likelihood of being diagnosed with AS (OR: 1.02; 95% CI: 1.02-1.02). When stratifying by race, Black beneficiaries living in a high-segregation county, compared to a low/moderate-segregation county, had a lower likelihood of AS diagnosis (OR: 0.97; 95% CI 0.96-0.98). In contrast, White beneficiaries living in a high-segregation county, compared to a low/moderate-segregation county, had a higher likelihood of AS diagnosis (OR: 1.02; 95% CI: 1.02-1.03) as seen in Figure 1. The interaction between race and county-level segregation with AS diagnosis was significant (*P* < 0.0001).

**Table 2:** Annual Rate of AS Diagnosis and AVR Per 100,000 Patient-Years

	Overall	Low/Moderate Segregation (SI <60)	High Segregation (SI ≥60)	*P* Value
AS diagnosis	1,083	1,065	1,149	<0.001
TAVI	107	108	104	<0.001
SAVR	73	73	70	<0.001
Any AVR	180	181	174	<0.001

Values are rates per 100,000 years

AS = aortic stenosis; AVR = aortic valve replacement; SAVR = surgical aortic valve replacement; SI = segregation index; TAVI = transcatheter aortic valve intervention.

After multivariable adjustment, race was associated with AS diagnosis; Black beneficiaries, compared to White beneficiaries had lower rates of AS diagnosis (OR: 0.51; 95% CI: 0.50-0.52). This association was attenuated, but persisted, among Black beneficiaries living in a low/moderate-segregation county, as compared with the association observed among those living in a high-segregation county (Supplemental Figure 1).

### Aortic valve replacement

Annual rates of AVR per 100,000 patient-years can be found in Table 2. Among those with an established diagnosis of AS, 145,764 (17%) underwent any AVR. Of those, 95,944 (66%) underwent TAVI, and 49,820 (34%) underwent SAVR. After multivariable adjustment among all beneficiaries, living in a high-segregation county, compared with a low/moderate-segregation county, was weakly associated with a decreased likelihood of TAVI (OR: 0.99; 95% CI: 0.98-1.00) and an increased likelihood of SAVR (OR: 1.01; 95% CI: 1.00-1.02), but no difference in any AVR (OR: 1.00; 95% CI: 1.00-1.01). In adjusted models stratified by race, Black beneficiaries living in a high-segregation county, compared to a low/moderate-segregation county, had lower rates of TAVI (OR: 0.89; 95% CI: 0.86-0.93) and any AVR (OR: 0.94; 95% CI: 0.91-0.97), but no differences in rates of SAVR (OR: 1.01; 95% CI: 1.00-1.02). In contrast, White beneficiaries living in a high-segregation county, compared to a low/moderate-segregation county, had no differences in rates of TAVI, SAVR, or any AVR (Figure 1). The interaction between race and county-level segregation was significant for TAVI, but not for SAVR or any AVR.

After multivariable adjustment, Black beneficiaries, compared to White beneficiaries, were associated with lower rates of TAVI (OR: 0.35; 95% CI: 0.34-0.37), SAVR (OR: 0.36; 95% CI: 0.35-0.38), and any AVR (OR: 0.36; 95% CI: 0.35-0.38). The association between Black beneficiaries and lower rates of TAVI was attenuated, but persisted, among patients living in a low/moderate-segregation county, as compared to the association observed among those living in a high-segregation county (Supplemental Figure 1).

### 30-day outcomes post-AVR

The primary 30-day composite outcome (mortality, readmission, stroke), occurred in 14.6% of those who received TAVI, 17.5% of those who received SAVR, and 15.7% of those who received any AVR (Table 3). After multivariable adjustment among White patients who received TAVI, living in a high-segregation county, compared to a low/moderate-segregation county, was associated with decreased likelihood of the 30-day composite outcome driven by a decrease in readmission, but no difference in mortality or stroke. Among Black patients, there was no association between county segregation and 30-day outcomes. Among White patients undergoing SAVR, living in a high-segregation county was associated with decreased 30-day mortality and stroke. This association was not present among Black patients receiving SAVR (Table 4).

**Table 3:** 30-Day Outcomes Post-AVR

	Overall	Low/Moderate Segregation (SI <60)	High Segregation (SI ≥60)	*P* Value
Composite	14.6	14.7	14.4	0.33
Mortality	2.02	2.03	2.00	0.80
Readmission	13.2	13.3	13.0	0.26
Stroke	0.42	0.44	0.35	0.06
Composite	17.5	17.6	17.4	0.62
Mortality	3.80	3.85	3.61	0.22
Readmission	14.6	14.6	14.5	0.74
Stroke	0.36	0.33	0.47	<0.01
Composite	15.7	15.8	15.6	0.24
Mortality	2.72	2.75	2.62	0.22
Readmission	13.7	13.8	13.6	0.25
Stroke	0.40	0.40	0.39	0.96
Urgent/emergent admission	16.5	16.1	18.0	<0.001
Transfemoral access	98.6	98.6	98.7	0.26

Values are percentages.

AVR = aortic valve replacement.

**Table 4:** Association Between High-Segregation^a^ and 30-Day Outcomes Post-AVR Stratified by Race

	Black			White		
	aOR	95% CI	*P* Value	aOR	95% CI	*P* Value
TAVI						
30-d all-cause mortality, and all-cause readmission ischemic stroke	1.07	0.88-1.32	0.49	0.95	0.91-0.99	0.01
30-d all-cause mortality	0.97	0.53-1.79	0.92	0.95	0.86-1.06	0.40
30-d all-cause readmission	1.07	0.87-1.33	0.52	0.94	0.90-0.99	0.01
30-d ischemic stroke as defined by ICD-10 codes	0.46	0.12-1.71	0.25	0.78	0.60-1.01	0.06
SAVR						
30-d all-cause mortality, and all-cause readmission ischemic stroke	1.03	0.84-1.26	0.81	0.96	0.91-1.00	0.08
30-d all-cause mortality	0.81	0.52-1.24	0.33	0.90	0.81-1.00	0.05
30-d all-cause readmission	1.09	0.86-1.37	0.48	0.96	0.91-1.01	0.15
30-d ischemic stroke as defined by ICD-10 codes	0.25	0.03-2.53	0.24	1.46	1.08-1.98	0.01
Any AVR						
30-d all-cause mortality, and all-cause readmission ischemic stroke	1.07	0.93-1.24	0.33	0.96	0.93-0.99	0.01
30-d all-cause mortality	0.90	0.64-1.27	0.54	0.93	0.87-1.01	0.08
30-d all-cause readmission	1.10	0.94-1.29	0.23	0.95	0.92-0.99	0.01
30-d ischemic stroke as defined by ICD-10 codes	0.47	0.16-1.37	0.17	0.99	0.82-1.21	0.94

Models are adjusted for demographic factors and comorbidities.

aOR = adjusted odds ratio; AVR = aortic valve replacement; SAVR = surgical aortic valve replacement; TAVI = transcatheter aortic valve intervention.

a Ref: Low/moderate segregation.

Black beneficiaries were not independently associated with 30-day outcomes (Supplemental Table 3). There were no significant interactions between Black beneficiaries and high-segregation residence on 30-day outcomes (*P* > 0.05 for all outcomes).

### Sensitivity analysis

In adjusted models using SI as a discrete variable composed of 10-point increments, among Black individuals, increasing segregation was associated with decreased rates of AS diagnosis, TAVI and any AVR, but no difference in SAVR (Supplemental Table 4). In adjusted models for the subset of individuals with an established diagnosis of AS, a similar association to that of the overall cohort was observed between segregation and race with TAVI, SAVR, and any AVR (Supplemental Figures 2 and 3).

## Discussion

In this national study, we assessed the association of Black-White residential segregation and individual-level race with AS diagnosis, management, and outcomes among Medicare FFS beneficiaries. We found that, compared to White adults, Black adults overall were 49% less likely to be diagnosed with AS and 65% less likely to undergo TAVI but had similar outcomes once TAVI was performed. Furthermore, we found that in high-segregation counties, compared to low/moderate-segregation counties, Black adults were less likely to be diagnosed with AS and receive TAVI whereas White adults were more likely to be diagnosed with AS but had no difference in rates of TAVI (Central Illustration).

**Central Illustration:**
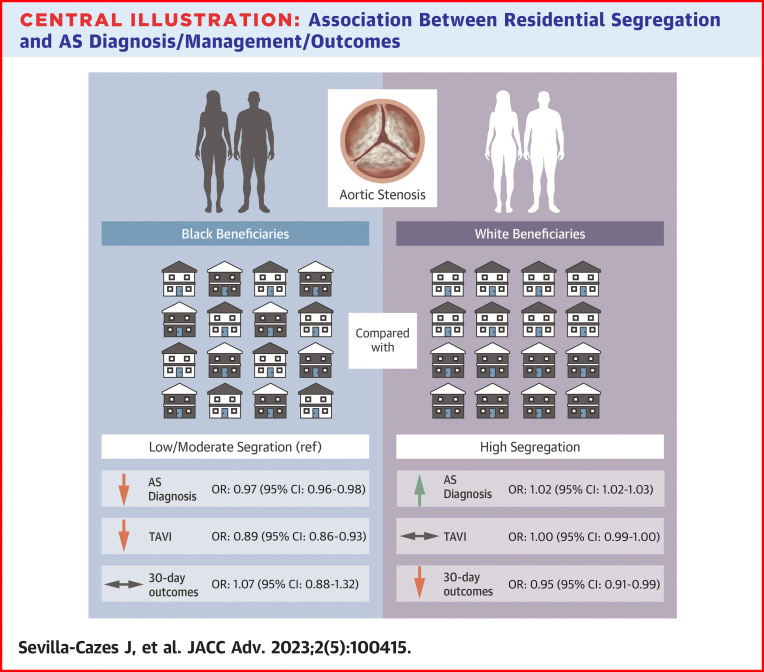
**Association Between Residential Segregation and AS Diagnosis/Management/Outcomes** Black people living in high-segregation counties, compared to low/moderate-segregation counties, have a lower likelihood of aortic stenosis diagnosis and transcatheter aortic valve implantation, but no difference in 30-day outcomes. In contrast, White people living in high-segregation counties, compared to low/moderate-segregation counties, have higher likelihood of aortic stenosis diagnosis, no difference in transcatheter aortic valve implantation, and improved 30-day outcomes. AS = aortic stenosis; OR = odds ratio; ref = reference; TAVI = transcatheter aortic valve intervention.

Black-White disparities in the diagnosis and management of AS have been previously described^11^^,^^25^; however, addressing these disparities effectively requires a more complete understanding of their multifactorial etiology. While racial residential segregation, a common form of systemic racism, has been identified as a fundamental cause of Black-White health disparities,^26^ this study is the first to explore its association with AS care. Residence in segregated neighborhoods is closely correlated with poverty and disproportionately affects Black people independent of their income.^27^ Living in segregated neighborhoods has been found to be associated with a higher prevalence of risk factors for the progression of AS, an increased risk of developing cardiovascular disease, and mortality.^6^, ^7^, ^8^, ^9^^,^^25^ Racial segregation has also been found to be associated with decreased access to health care, which may also lead to the decreased detection and treatment of severe AS.^28^ Our study found an increased diagnosis of AS among White individuals in high-segregation counties, and decreased diagnosis of AS and treatment with TAVI among Black individuals in high-segregation. Viewed through the lens of intersectionality, our findings suggests that race and residence in a segregated county intersect in a cumulative way that results in Black adults living in high-segregation counties having the highest risk of decreased access to AS care and resultant underdetection and undertreatment of disease.

Our study identifies an intersectional disparity in the diagnosis and management of AS, in which there is an increased diagnosis of AS among White individuals in high-segregation counties, and decreased diagnosis of AS and treatment with TAVI among Black individuals in high-segregation. This suggests Black adults living in high-segregation counties are a population at high risk of decreased access to AS care and resultant underdetection and undertreatment of disease.

A useful framework to understand the factors contributing to our findings is proposed by Batchelor et al,^2^ who categorized the barriers contributing to AS disparities into patient-related factors, health care/system factors, and disease related factors. Previous studies have found a lower prevalence of AS among Black individuals; however, the absence of a compelling biological mechanism to explain racial differences in the development and progression of AS raises the possibility of bias in these observations and underscores the importance that patient and health care factors may have on AS diagnosis and management.^2^^,^^20^ Among patient-related factors, lower rates of insurance coverage—including higher rate of uninsured and lower rate of private health coverage—among Black people may result in decreased rates of disease identification, surveillance, and treatment; however, racial disparities in health care access remain after controlling for differences in insurance coverage.^29^ For Black individuals that are insured and able to access care, cultural differences and distrust in the medical system may cause this population to be less likely to seek medical care and can shape their medical decision-making.^2^^,^^30^ This distrust may be further increased by physician factors such as lack of culturally competent care and subconscious bias.^31^ Furthermore, competing demands, particularly among those living in high-segregation counties, such as housing instability, food insecurity, and lack of job flexibility, can lead to delayed care or refusal to pursue interventions once offered.^32^^,^^33^

Among health care–system factors, Black individuals have been found to receive lower quality primary care, be less likely to have board-certified primary care providers, and be less likely to have access to high quality diagnostic imaging,^34^ which in turn may preclude initial diagnosis of AS. Once diagnosed with valvular disease, Black patients are less likely to have guideline-recommended surveillance echocardiograms^35^ and to be referred to a cardiologist or a cardiac surgeon.^32^^,^^36^^,^^37^ While Black patients tend to live in closer geographic proximity to higher-quality hospitals than White patients do, they are more likely to receive surgery at low-quality hospitals.^38^ Furthermore, metropolitan zip codes with high proportion Black patients have been found to have lower rates of TAVI despite having geographic proximity to TAVI-capable hospitals.^39^ It is likely that these factors are further exacerbated among those living in high-segregation counties as their options may be limited to receiving care at hospitals primarily serving people of color which are more likely to be under resourced.^10^ These factors likely have a cumulative effect as evidence by our finding of a larger magnitude association between residence in a high-segregation county and TAVI than with AS diagnosis.

For Black patients who received TAVI, the interaction between county-level racial segregation and individual-level race was not associated with 30-day outcomes. However, among White patients, living in a high-segregation county, was associated with a decreased likelihood of 30-day readmission, but no difference in mortality or stroke. The cause of this unexpected finding—given a higher prevalence of risk factors traditionally associated with readmission among this population—is unclear. Studies to further understand these patterns are warranted.

Our study identifies Black people living in high-segregation counties as a population at high risk of underdiagnosis and undertreatment of AS, but with similar outcomes once TAVR is performed. This disparity likely reflects the deleterious effect of systemic racism on health care access and surveillance of disease in Black communities in the United States. At the individual level, community based interventions, such as patient navigator programs, which have been effective at ameliorating disparities in colorectal and breast cancer screening, represent a promising way to reduce disparities in the short term.^40^ However, mitigating this disparity over the long term will require deeper system reform targeting individuals, communities, health care providers, and institutions.^41^

### Study limitations

Our study was subject to several limitations. First, our study focused on 1 form of structural racism and is unable to assess the degree to which other forms of racism may be contributing to disparities in AS diagnosis and management. Second, we identified patients with AS using diagnostic codes which are highly specific but poorly sensitive and it is likely that we misclassified some beneficiaries resulting in a significant underestimation of disease prevalence. Third, we were unable to determine severity of AS at the time of diagnosis. Fourth, while our models were adjusted for demographic and clinical characteristics, there may still be unmeasured confounding. Sixth, many clinical factors which we were unable to measure go into the selection of TAVI vs SAVR. Seventh, we determined exposure to a high-segregation county based on county at the time of Medicare FFS enrollment and are unable to account for the cumulative effects of living in a high-segregation county which can persist after moving to a less segregated county.^6^ Eight, our cohort included only Medicare FFS beneficiaries; we are unable to assess the role of health insurance, or generalize our findings to individuals with Medicare advantage, private insurance, or the uninsured. Finally, we focused our analysis on Black-White disparities, and we did not assess the mechanisms driving disparities in AS diagnosis and TAVI among other historically disadvantaged populations.

## Conclusions

In this Medicare FFS population, we found that living in a high-segregation county was independently associated with decreased odds of AS diagnosis and TAVI for Black, but not White, individuals. This association is likely driven by patient-related and health care–system factors that result in decreased access to quality AS care among Black people living in high-segregation counties. Among individuals who have received TAVI, race was not associated with clinical outcomes. Our study adds to evidence that racial residential segregation is an important form of structural racism which may have a significant impact on the diagnosis and management of AS. Devoting resources to address structural racism as a social determinant of health is critical to addressing disparities in cardiovascular care.

PERSPECTIVES **COMPETENCY IN SYSTEMS-BASED PRACTICE:** Black people living in high-segregation counties represent a high-risk population with significant barriers to diagnosis and treatment of AS.

**TRANSLATIONAL OUTLOOK:** Further research is needed to define the mechanisms by which residential segregation affects the diagnosis and treatment of AS among Black people and identify targets for intervention.

## Funding support and author disclosures

This study was funded by 10.13039/100004374Medtronic. The study design, data analysis and manuscript preparation was performed independently by the investigator team without input from the funder. Dr Almarzooq has received research grant support from the Kuwait Foundation for the Advancement of Science. Dr Strom has received grant funding from Edwards Lifesciences, Anumana, Ultromics, EchoIQ, and HeartSciences; consulting for Bracco Diagnostics and GE Healthcare; speaker fees from Northwest Imaging Forums; and is on the Scientific Advisory Board for Edwards Lifesciences and EchoIQ. Dr Keller is a speaker and proctor for Medtronic Corporation, Edwards Lifesciences, and Stryker Corporation. Dr Wadhera has received consulting fees from CVS Health and Abbott outside the submitted work. Dr Yeh has received grant funding and consulting fees from Abbott Vascular, Boston Scientific, and Medtronic. All other authors have reported that they have no relationships relevant to the contents of this paper to disclose.

## References

[1] Otto C.M., Nishimura R.A., Bonow R.O. (2021). 2020 ACC/AHA guideline for the management of patients with valvular heart disease: a report of the American College of Cardiology/American Heart Association Joint Committee on Clinical Practice Guidelines. J Am Coll Cardiol.

[2] Batchelor W., Anwaruddin S., Ross L. (2019). Aortic valve stenosis treatment disparities in the underserved: JACC Council perspectives. J Am Coll Cardiol.

[3] Wilson J.B., Jackson L.R., Ugowe F.E. (2020). Racial and ethnic differences in treatment and outcomes of severe aortic stenosis: a review. J Am Coll Cardiol Intv.

[4] Braveman P., Egerter S., Williams D.R. (2011). The social determinants of health: coming of age. Annu Rev Public Health.

[5] Churchwell K., Elkind M.S.V., Benjamin R.M. (2020). Call to action: structural racism as a fundamental driver of health disparities: a presidential advisory from the American Heart Association. Circulation.

[6] Reddy N.M., Mayne S.L., Pool L.R. (2022). Exposure to neighborhood-level racial residential segregation in young adulthood to midlife and incident subclinical atherosclerosis in Black adults: the coronary artery risk development in young adults study. Circ Cardiovasc Qual Outcomes.

[7] Greer S., Kramer M.R., Cook-Smith J.N., Casper M.L. (2014). Metropolitan racial residential segregation, and cardiovascular mortality: exploring pathways. J Urban Health.

[8] Kershaw K.N., Osypuk T.L., Do D.P., De Chavez P.J., Diez Roux A.V. (2015). Neighborhood-level racial/ethnic residential segregation and incident cardiovascular disease: the multi-ethnic study of atherosclerosis. Circulation.

[9] Kyalwazi A.N., Loccoh E.C., Brewer L.C. (2022). Disparities in cardiovascular mortality between Black and White adults in the United States, 1999 to 2019. Circulation.

[10] Himmelstein G., Himmelstein K.E.W. (2020). Inequality set in concrete: physical resources available for care at hospitals serving people of color and other U.S. hospitals. Int J Health Serv.

[11] Alkhouli M., Holmes D.R., Carroll J.D. (2019). Racial disparities in the utilization and outcomes of TAVR: TVT Registry report. J Am Coll Cardiol Intv.

[12] Kundi H., Faridi K.F., Wang Y. (2019). Geographic patterns of growth for transcatheter aortic valve replacement in the United States. Circulation.

[13] US Census Bureau Delineating metropolitan and micropolitan statistical areas. https://www.census.gov/programs-surveys/metro-micro/about.html.

[14] County Health Rankings Residential segregation – Black/White. https://www.countyhealthrankings.org/explore-health-rankings/measures-data-sources/county-health-rankings-model/health-factors/social-and-economic-factors/family-social-support/residential-segregation-blackwhite.

[15] Wadhera R.K., Wang Y., Figueroa J.F., Dominici F., Yeh R.W., Joynt Maddox K.E. (2020). Mortality and hospitalizations for dually enrolled and nondually enrolled Medicare beneficiaries aged 65 years or older, 2004 to 2017. JAMA.

[16] Chronic Conditions Data Warehouse Chronic conditions. https://www2.ccwdata.org/web/guest/condition-categories-chronic#cc27.

[17] Chronic Conditions Data Warehouse Other chronic health, mental health, and potentially disabling conditions. https://www2.ccwdata.org/web/guest/condition-categories-other.

[18] White M.J. (1986). Segregation and diversity measures in population distribution. Popul Index.

[19] Massey D.S., Denton N.A. (1989). Hypersegregation in U.S. metropolitan areas: Black and Hispanic segregation along five dimensions. Demography.

[20] Thanassoulis G., Campbell C.Y., Owens D.S. (2013). Genetic associations with valvular calcification and aortic stenosis. N Engl J Med.

[21] Strom J.B., Xu J., Sun T. (2022). Characterizing the accuracy of ICD-10 administrative claims for aortic valve disease. Circ Cardiovasc Qual Outcomes.

[22] Strom J.B., Tamez H., Zhao Y. (2019). Validating the use of registries and claims data to support randomized trials: rationale and design of the extending trial-based evaluations of medical therapies using novel sources of data (EXTEND) study. Am Heart J.

[23] Almarzooq Z.I., Kazi D.S., Wang Y. (2022). Outcomes of stroke events during transcatheter aortic valve implantation. EuroIntervention.

[24] von Elm E., Altman D.G., Egger M. (2007). The strengthening the reporting of observational studies in epidemiology (STROBE) statement: guidelines for reporting observational studies. PLoS Med.

[25] Freeman R.V., Otto C.M. (2005). Spectrum of calcific aortic valve disease: pathogenesis, disease progression, and treatment strategies. Circulation.

[26] Williams D.R., Collins C. (2001). Racial residential segregation: a fundamental cause of racial disparities in health. Public Health Rep.

[27] Logan J.R. (2011). Separate and unequal: the neighborhood gap for Blacks, Hispanics, and Asians in metropolitan America. http://www.s4.brown.edu/us2010/Data/Report/report0727.pdf.

[28] Caldwell J.T., Ford C.L., Wallace S.P., Wang M.C., Takahashi L.M. (2017). Racial and ethnic residential segregation and access to health care in rural areas. Health Place.

[29] Dickman S.L., Gaffney A., McGregor A. (2022). Trends in health care use among Black and White persons in the US, 1963-2019. JAMA Netw Open.

[30] Noonan A.S., Velasco-Mondragon H.E., Wagner F.A. (2016). Improving the health of African Americans in the USA: an overdue opportunity for social justice. Public Health Rev.

[31] Breathett K., Jones J., Lum H.D. (2018). Factors related to physician clinical decision-making for African-American and Hispanic patients: a qualitative meta-synthesis. J Racial Ethn Health Disparities.

[32] Sleder A., Tackett S., Cerasale M. (2017). Socioeconomic and racial disparities: a case-control study of patients receiving transcatheter aortic valve replacement for severe aortic stenosis. J Racial Ethn Health Disparities.

[33] Yeung M., Kerrigan J., Sodhi S. (2013). Racial differences in rates of aortic valve replacement in patients with severe aortic stenosis. Am J Cardiol.

[34] Bach P.B., Pham H.H., Schrag D., Tate R.C., Hargraves J.L. (2004). Primary care physicians who treat Blacks and Whites. N Engl J Med.

[35] Tanguturi V.K., Bhambhani V., Picard M.H., Armstrong K., Wasfy J.H. (2019). Echocardiographic surveillance of valvular heart disease in different sociodemographic groups. J Am Coll Cardiol Img.

[36] Cook N.L., Ayanian J.Z., Orav E.J., Hicks L.S. (2009). Differences in specialist consultations for cardiovascular disease by race, ethnicity, gender, insurance status, and site of primary care. Circulation.

[37] Cruz Rodriguez B., Acharya P., Salazar-Fields C., Horne A. (2017). Comparison of frequency of referral to cardiothoracic surgery for aortic valve disease in Blacks, Hispanics, and Whites. Am J Cardiol.

[38] Dimick J., Ruhter J., Sarrazin M.V., Birkmeyer J.D. (2013). Black patients more likely than Whites to undergo surgery at low-quality hospitals in segregated regions. Health Aff.

[39] Nathan A.S., Yang L., Yang N. (2022). Racial, ethnic, and socioeconomic disparities in access to transcatheter aortic valve replacement within major metropolitan areas. JAMA Cardiol.

[40] Neal C.D., Weaver D.T., Raphel T.J. (2018). Patient navigation to improve cancer screening in underserved populations: reported experiences, opportunities, and challenges. J Am Coll Radiol.

[41] Bailey Z.D., Krieger N., Agénor M., Graves J., Linos N., Bassett M.T. (2017). Structural racism and health inequities in the USA: evidence and interventions. Lancet.

