# Supplementary material for: Residential Racial Segregation in Aortic Stenosis Diagnosis and Transcatheter Aortic Valve Implantation Among Medicare Patients
Source: JACC Adv. 2023 Jul 19;2(5):100415. doi: 10.1016/j.jacadv.2023.100415 (PMC11198501; doi:10.1016/j.jacadv.2023.100415)
Supplement: Supplementary Materials [file mmc1.docx]

| **Supplemental Table 1: Performance of ICD-10 Claims to Identify AS on Paired TTE** | | | | | | | |
| --- | --- | --- | --- | --- | --- | --- | --- |
| **Counties** | **Sensitivity (%)** | **Specificity (%)** | **NPV (%)** | **PPV (%)** | **+LR** | **-LR** | **Kappa Statistic** |
|  |  |  |  |  |  |  |  |
| All | 49.5  (46.8, 52.2) | 96.3  (95.6, 96.8) | 85.8  (84.8, 86.8) | 80.7  (77.9, 83.3) | 13.26  (11.3, 15.6) | 0.53  (0.5, 0.6) | 0.53  (0.50-0.56) |
| Low/  Moderate Segregation (SI<60) | 48.7  (45.2, 52.3) | 96.4  (95.6, 97.0) | 86.0  (85.2, 86.8) | 80.3  (76.8, 83.4) | 13.4  (10.8, 16.5) | 0.5  (0.5, 0.6) | 0.52  (0.49, 0.56) |
| High Segregation (SI>=60) | 50.8  (46.3, 55.2) | 96.0  (94.8, 96.9) | 85.2  (84.0, 86.3) | 81.0  (76.6, 84.7) | 12.6  (9.7, 16.4) | 0.5  (0.5, 0.6) | 0.53  (0.49, 0.58) |
| Diagnostic performance and 95% confidence intervals of ICD-10 Claims (I35.0, I35.2, I06.0, I06.2, Q23.0) to identify any AS on TTE are presented for the overall population and stratified by county segregation. Kappa statistic for agreement between claims and TTE ascertainment of AS are provided. AS=aortic stenosis, ICD-10= International Classification of Disease, Tenth Revision, +LR=positive likelihood ratio, -LR=negative likelihood ratio, NPV=negative predictive value, PPV=positive predictive value, SI=segregation index, TTE=transthoracic echocardiogram, | | | | | | | |

| Supplemental Table 2. Baseline Characteristics of Patients with an AS Diagnosis stratified by segregation | | | | |
| --- | --- | --- | --- | --- |
|  | Overall | Low/Moderate  Segregation  (SI <60) | High Segregation (SI >= 60) | p-value |
| Total (n) | 864512 | 664187 | 200325 |  |
| Age | 81.3 (8.2) | 81.2 ( 8.1) | 81.8 (8.2) | <0.0001 |
| Female | 50 | 49.4 | 51.9 | <0.0001 |
| Black | 4.9 | 4.2 | 7.2 | <0.0001 |
| Dual Enrollment | 9.8 | 9.3 | 11.5 | <0.0001 |
| Clinical Comorbidities | | | | |
| Myocardial Infarction | 0.8 | 0.8 | 0.8 | 0.80 |
| Atrial Fibrillation | 12.5 | 12.7 | 12.1 | <0.0001 |
| Chronic Kidney Disease | 22.3 | 22.6 | 21.6 | <0.0001 |
| Chronic Obstructive Pulmonary Disease | 10.5 | 10.6 | 10.2 | <0.0001 |
| Congestive Heart Failure | 18 | 17.9 | 18.4 | <0.0001 |
| Diabetes | 23.2 | 23.2 | 23.1 | 0.57 |
| Ischemic Heart Disease | 31.1 | 31.3 | 30.4 | <.0001 |
| Stroke/Transient Ischemic Attack | 3.5 | 3.6 | 3.5 | 0.42 |
| Cancer | 7.3 | 7.3 | 7.2 | 0.53 |
| Hyperlipidemia | 38.9 | 39.4 | 37.2 | <0.0001 |
| Hypertension | 48.2 | 48.6 | 46.9 | <0.0001 |
| Obesity | 19.3 | 19.4 | 19.3 | 0.40 |
| Peripheral Vascular Disease | 25.1 | 24.6 | 26.9 | <0.0001 |
| Aortic Valve Replacement | | | | |
| TAVI | 6.2 | 6.4 | 5.8 | <0.0001 |
| SAVR | 4.3 | 4.4 | 3.8 | <0.0001 |
| Any AVR | 10.5 | 10.7 | 9.6 | <0.0001 |
| Age is presented as mean (standard deviation), all other values are presented as percentages.  AS=aortic stenosis, AVR=aortic valve replacement, SAVR= surgical aortic valve replacement, SI= segregation index, TAVI=transcatheter aortic valve intervention | | | | |

| **Supplemental Table 3. Association Between Race and 30-day Outcomes post-AVR** | | | |
| --- | --- | --- | --- |
|  | Reference: White | | |
|  | aOR | CI | p-value |
| **TAVI** | | | |
| 30-day Composite | 0.99 | 0.99 - 1.09 | 0.85 |
| 30-day Mortality | 0.88 | 0.88 - 1.16 | 0.39 |
| 30-day Readmission | 0.99 | 0.90 - 1.09 | 0.85 |
| 30-day Stroke | 1.32 | 0.79 - 2.19 | 0.29 |
| **SAVR** | | | |
| 30-day Composite | 1.08 | 0.98 - 1.20 | 0.11 |
| 30-day Mortality | 1.21 | 0.99- 1.47 | 0.06 |
| 30-day Readmission | 1.04 | 0.93 - 1.16 | 0.53 |
| 30-day Stroke | 1.19 | 0.60 - 2.36 | 0.62 |
| **Any AVR** | | | |
| 30-day Composite | 1.04 | 0.97 - 1.12 | 0.24 |
| 30-day Mortality | 1.11 | 0.94- 1.30 | 0.22 |
| 30-day Readmission | 1.02 | 0.94 - 1.09 | 0.69 |
| 30-day Stroke | 1.27 | 0.85 - 1.91 | 0.25 |
| Models are adjusted for demographic factors and comorbidities  AVR=aortic valve replacement, aOR=adjusted odds ratio, CI = confidence interval, SAVR= surgical aortic valve replacement, TAVI=transcatheter aortic valve intervention | | | |

| **Supplemental Table 4. Association Between 10-point increments in segregation and AS Diagnosis/Management** | | | | |
| --- | --- | --- | --- | --- |
|  |  | aOR | 95% CI | p-value |
| AS Diagnosis | Black | 0.99 | 0.98-0.99 | <0.0001 |
|  | White | 1.03 | 1.03-1.04 | <0.0001 |
| TAVI | Black | 0.94 | 0.92-0.96 | <0.0001 |
|  | White | 1.01 | 1.01-1.02 | <0.0001 |
| SAVR | Black | 1.00 | 0.97-1.03 | 0.97 |
|  | White | 1.01 | 1.01-1.02 | <0.0001 |
| Any AVR | Black | 0.96 | 0.95-0.98 | 0.0002 |
|  | White | 1.02 | 1.01-1.02 | <0.0001 |
| Models are adjusted for demographic factors and comorbidities  AVR=aortic valve replacement, aOR= adjusted odds ratio, CI = confidence interval, SAVR= surgical aortic valve replacement, TAVI=transcatheter aortic valve intervention | | | | |

Supplemental Figure 1

TITLE: Association between race and AS diagnosis/management stratified by county segregation.





CAPTION: A mixed-effect regression was used to model the rates of AS diagnosis, TAVI, SAVR, and any AVR, as a function of race adjusting for beneficiary demographic and clinical characteristics. Black race, compared to White race, was associated with a lower likelihood of AS diagnosis, TAVI, and any AVR. This association persisted among those living in a low/moderate-segregation county.

AS=aortic stenosis, AVR=aortic valve replacement, CI=confidence interval Interaction = Black race * high-segregation interaction variable, aOR=adjusted odds ratio, SAVR=surgical aortic valve replacement, TAVI=transcatheter aortic valve intervention.

Supplemental Figure 2

TITLE: Association between county segregation and AVR among AS patients


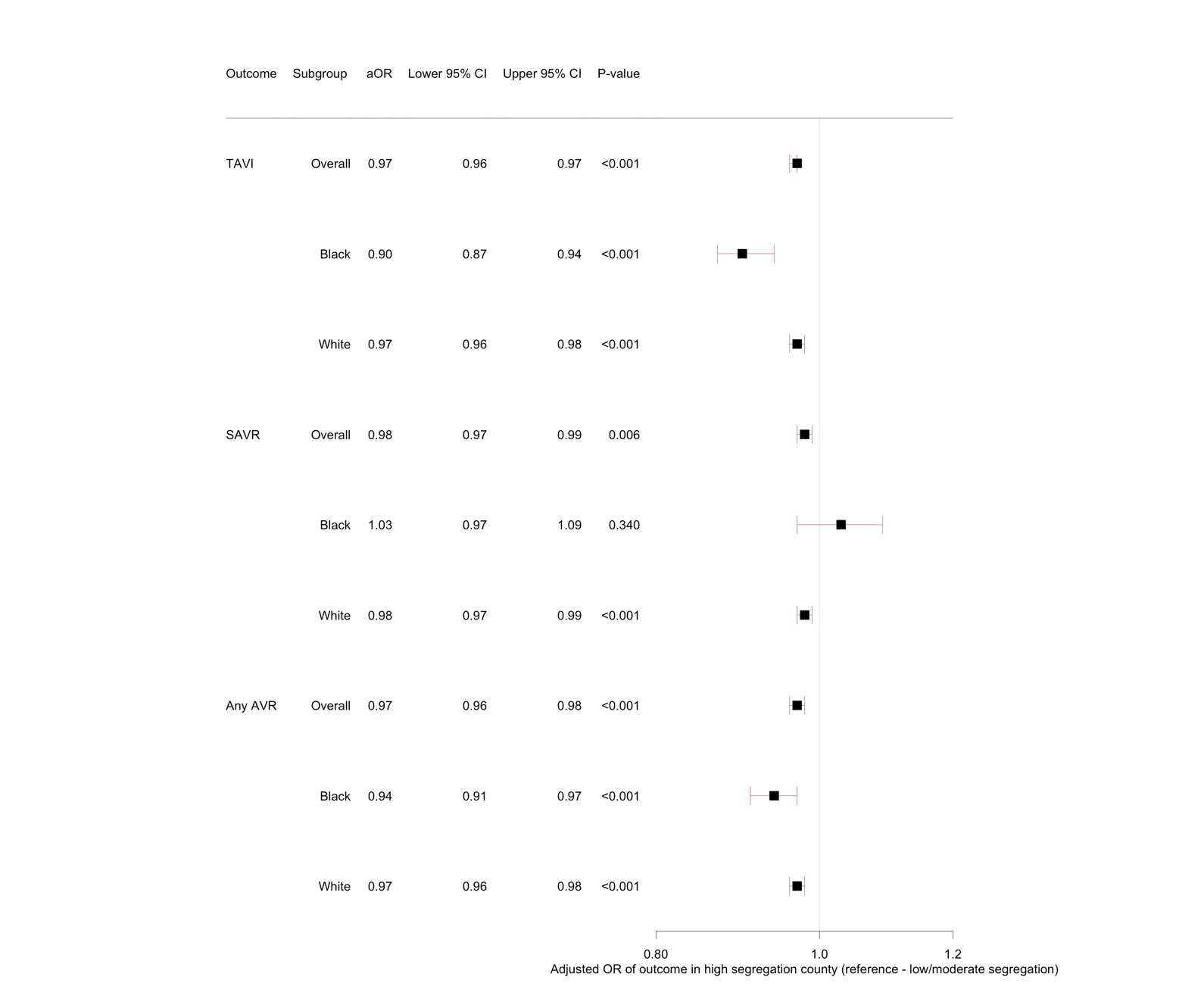


CAPTION: Among beneficiaries with an established AS diagnosis, mixed-effect regression was used to model the rates of TAVI, SAVR, and any AVR, as a function of county segregation adjusting for beneficiary demographic and clinical characteristics. For both Black and White patients, living in a high-segregation county, compared to a low/moderate-segregation county, was associated with lower likelihood TAVI. AS=aortic stenosis, aOR=adjusted odds ratio, AVR=aortic valve replacement, CI=confidence interval, SAVR=surgical aortic valve replacement, TAVI=transcatheter aortic valve intervention.

Supplemental Figure 3

TITLE: Association between race and AVR among AS patients

**
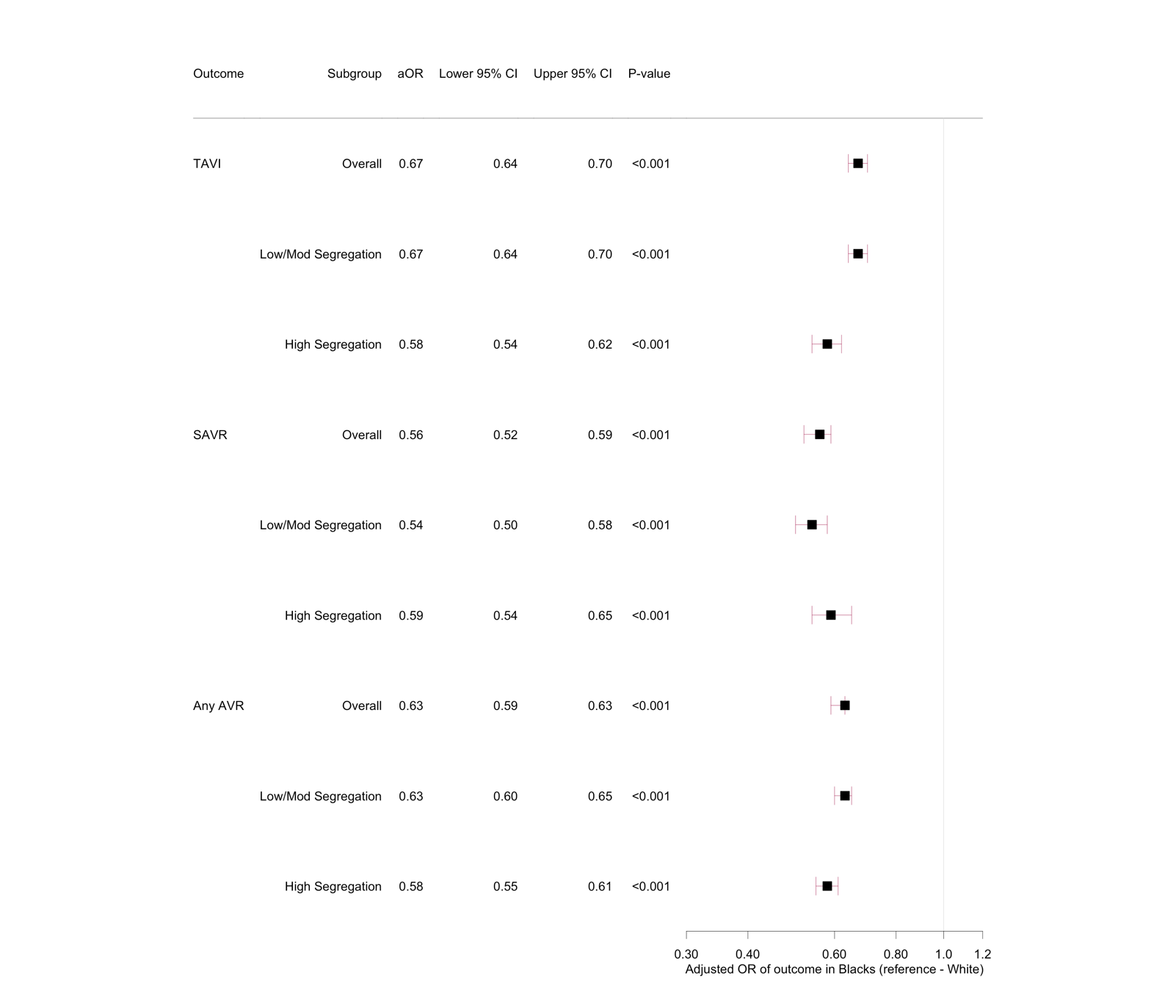
**

CAPTION: Among beneficiaries with an established AS diagnosis, mixed-effect regression was used to model the rates of TAVI, SAVR, and any AVR, as a function of race adjusting for beneficiary demographic and clinical characteristics. Black race, compared to White race, was associated with lower likelihood of TAVI. AS=aortic stenosis, aOR=adjusted odds ratio, AVR=aortic valve replacement, CI=confidence interval, SAVR=surgical aortic valve replacement, TAVI=transcatheter aortic valve intervention.
